# Elevated co-expression of TIMM17A and NMT1 is associated with poor survival in non-small cell lung cancer

**DOI:** 10.1038/s41598-025-11897-9

**Published:** 2025-10-13

**Authors:** Alfred A. Chan, Kamya Sankar, Karen L. Reckamp, Begoña Díaz, Delphine J. Lee

**Affiliations:** 1 The Lundquist Institute for Biomedical Innovation at Harbor-UCLA Medical Center, 1124 West Carson Street, Torrance, CA 90502 USA; 2 Department of Medicine, Cedars-Sinai Medical Center, Los Angeles, CA USA; 3 Division of Hematology and Oncology, Department of Medicine, Harbor-UCLA Medical Center, Torrance, CA USA; 4 Jonsson Comprehensive Cancer Center, UCLA, Los Angeles, CA USA; 5 Department Medicine, David Geffen School of Medicine at UCLA, Los Angeles, CA USA

**Keywords:** The Cancer Genome Atlas, Lung adenocarcinoma, NMT1, TIMM17A, Survival, Computational biology and bioinformatics, Data mining, Transcriptomics, Non-small-cell lung cancer, Cancer genomics

## Abstract

Lung carcinoma cells harboring mutations in *STK11* and/or *KEAP1* in a *KRAS* mutant background have intrinsic therapeutic resistance. We found that these cells are sensitive to preclinical stage pharmacological inhibitors of N-myristoyltransferases, which reduce tumor growth in xenograft mouse models. Unexpectedly, the sensitivity to NMT inhibitors correlates with cell’s dependency on the inner mitochondrial protein Translocase of Inner Mitochondrial Membrane 17 A (Protein: TIM17A, Gene: *TIMM17A*). Leveraging data from The Cancer Genome Atlas (TCGA), we set to further explore the significance of N-myristoyltransferase-1 (*NMT1*) and *TIMM17A* expression individually and together in cox-regression models to test their association to clinical endpoints in lung carcinoma. Our results showed that lung adenocarcinoma (LUAD) tissue with high expression of both *NMT1* and *TIMM17A* had worse overall survival (OS), disease-specific survival (DSS), and disease-free survival (DFS) compared to those with low expression of both genes. Moreover, high *NMT1* was associated with worse OS, but only in the group with also high *TIMM17A*. This highlights a novel *NMT1*/*TIMM17A* axis as a promising pathway for predicting patient prognosis. Further studies are warranted investigating both genes as targets for novel therapeutic strategies for *KRAS* mutant non-small cell lung carcinoma with *STK11* and/or *KEAP1* co-mutations.

**Supplementary Information:**

The online version contains supplementary material available at 10.1038/s41598-025-11897-9.

## Introduction

The treatment paradigm for advanced non-small cell lung carcinoma (NSCLC) has significantly shifted in the last decade with the incorporation of checkpoint inhibitor immunotherapy and targeted therapies to treat tumors with specific molecular alterations. Despite these significant clinical advances, the median 5-year overall survival (OS) remains low at 25%^1^. The prognosis for advanced *KRAS*-mutant NSCLC is even worse and has remained poor for decades, with a median survival of 1.2 years^2^. *KRAS*-driven NSCLC is a distinct molecular subtype of NSCLC, that has been a historically challenging to therapeutically target, and even deemed “undruggable”^3^. Patients with *KRAS*-mutant NSCLC harboring co-mutations in *STK11* and/or *KEAP1* have been associated with the use of tobacco products and are notorious for being aggressive and resistant to treatment^4^. *STK11* and *KEAP1* mutations have been shown to confer primary resistance to anti-PD-L1/PD-1 immune checkpoint inhibitor (ICI) therapy in the presence of *KRAS* mutation^4^, although the combination with CTLA4 blockade shows promising results^5^. Despite *sotorasib* and *adagrasib* being approved for advanced NSCLC with *KRAS* G12C mutation in patients who have received at least one prior line of therapy^6^, outcomes remain poor. Therefore, ongoing efforts continue to test for combination therapies to overcome inhibitor resistance and to test for novel *KRAS* allele-specific inhibitors. The role of *STK11* and *KEAP1* co-occurring mutations on primary response rates, duration of response and evolution of resistance remains a principal area of ongoing research and an area of unmet need for new therapeutic options^7^.

We previously reported that NSCLC with *STK11* and/or *KEAP1* mutations in a *KRAS* mutant background (but not KRAS mutant cells lacking these co-mutations) are sensitive to inhibition of *N*-myristoyltransferases (NMTs)^8^. Preclinical stage inhibitors of NMTs (which target NMT1 and NMT2) have also shown efficacy against experimental models of lymphoma^9^ and are currently in stage 2 clinical trial^10^. We and others have shown that elevated mRNA expression of *NMT1* (the most abundant NMT enzyme in most tissues) correlates with decreased OS (overall survival) in lung carcinoma^11,12^.

We reported that NMT inhibition in lung carcinoma cells decreases the expression of the mitochondrial import protein TIM17A, but only those cells that are sensitive to NMT inhibition were also sensitive to loss of *TIMM17A* expression^12^. Accordingly, we found a significant correlation between sensitivity to NMT inhibition and TIM17A dependency in lung carcinoma cells, suggesting a link between both genes in lung adenocarcinoma^12^. We hypothesized that *NMT1-TIMM17A* is a novel vulnerability axis that may represent a specific target for therapy of lung carcinomas with *STK11* and/or *KEAP1* mutations in a *KRAS* mutant background. To further explore the unexpected relationship between NMT1 and TIM17A, we used data from The Cancer Genome Atlas (TCGA) to investigate the association of *NMT1* and *TIMM17A* mRNA transcript expression levels with overall survival (OS), disease-specific survival (DSS) and disease-free survival (DFS) in a lung adenocarcinoma (LUAD) cohort.

## Materials and methods

The Cancer Genome Atlas—Lung Adenocarcinoma (TCGA-LUAD) gene expression data and associated clinical data were queried from cBioPortal Pan Cancer Atlas. A total of 486 patients were linked to RNA-sequencing data (units: normalized counts). Demographic information included age, gender, smoking habits, patient performance status (ECOG, Karnofsky Score), and spirometry data. Clinical data included initial tissue weight, status of prior cancer diagnosis, primary tumor location, tumor pathological stage, and treatment administered such as radiation therapy or post-operative adjuvant therapy. The provided hypoxia scores had been calculated from transcriptomic data. The provided aneuploidy score, MSI Mantis Scores, MSI Sensor Score, total mutation, and total mutation burden score had been calculated from DNA mutation data. Comparing the distribution of continuous variables between groups (e.g. age distribution in high versus low *TIMM17A*) was performed using student *t*-test. Comparing the distribution of categorical variables between groups (e.g. gender distribution in high versus low *TIMM17A*) was performed using Fisher’s exact test.

Survival analysis was performed using Cox regression models. Association between clinical endpoints and gene expression data were assessed using hazard ratios (HR) and their 95% confidence intervals (CI). Gene expression data were tested as both a continuous variable (log-transformed and scaled) and a categorical variable (split dichotomously based on assessment as continuous variable). The complete model was adjusted for gender, age, tumor pathological stage, and *KRAS*/*STK11*/*KEAP1* mutation status. The fit for various steps of the multivariable modeling with terms added versus removed were tested using ANOVA.

To visualize how long a patient would survive for a given continuous value of gene expression, restricted mean survival (RMS) curves were used. More specifically, RMS curve plots the Cox-model estimated years of survival for every nth quantile of gene expression. Next, the normalized gene count values were dichotomized into high versus low expression groups using an optimal cutoff determined by log-rank tests evaluated at every 10th percentile. To visualize the percentage of patients surviving over time for the categories of low and high gene expression, Kaplan-Meier curves were used.

Validation dataset used lung adenocarcinoma cases from the Singapore Oncology Data Portal (OncoSG, *n* = 172 with OS-events = 44). Pre-processed transcriptomic data (STAR-aligned RSEM raw counts), mutational data as well as associated clinical and demographic data such as overall survival, tumor staging, age, gender, and smoking status were queried from their portal. By histological subtype, the majority of OncoSG lung cancers were acinar (87/172–49.3%) with some solid (18/172–9.9%) and papillary (19/172–11.7%) histological types; TCGA, on the other hand, were mostly either mixed (120/515–23.3%) or not specified (320/515–62.1%), leaving few that were acinar (18/515–3.5%) and solid (5/515–1.0%), and none that were labelled papillary. The OncoSG cohort consisted of individuals of East Asian Ancestry with fewer current smokers (36% vs. 85% in TCGA) and less overall mutational burden (92 vs. 312 in TCGA). Thus, for comparability to TCGA dataset, the OncoSG validation cohort analyzed only those who have ever-smoked (*n* = 62, OS-events = 20). Multivariable cox-regression models adjusted for age (mean ± SD: 64.1 ± 10.0), gender (n: Male = 49, Female = 13), and tumor stage (n: I = 35, II = 11, III = 13, IV = 1).

## Results

### High NMT1 and TIMM17A were associated with poor overall survival in lung adenocarcinoma

**Fig. 1:**
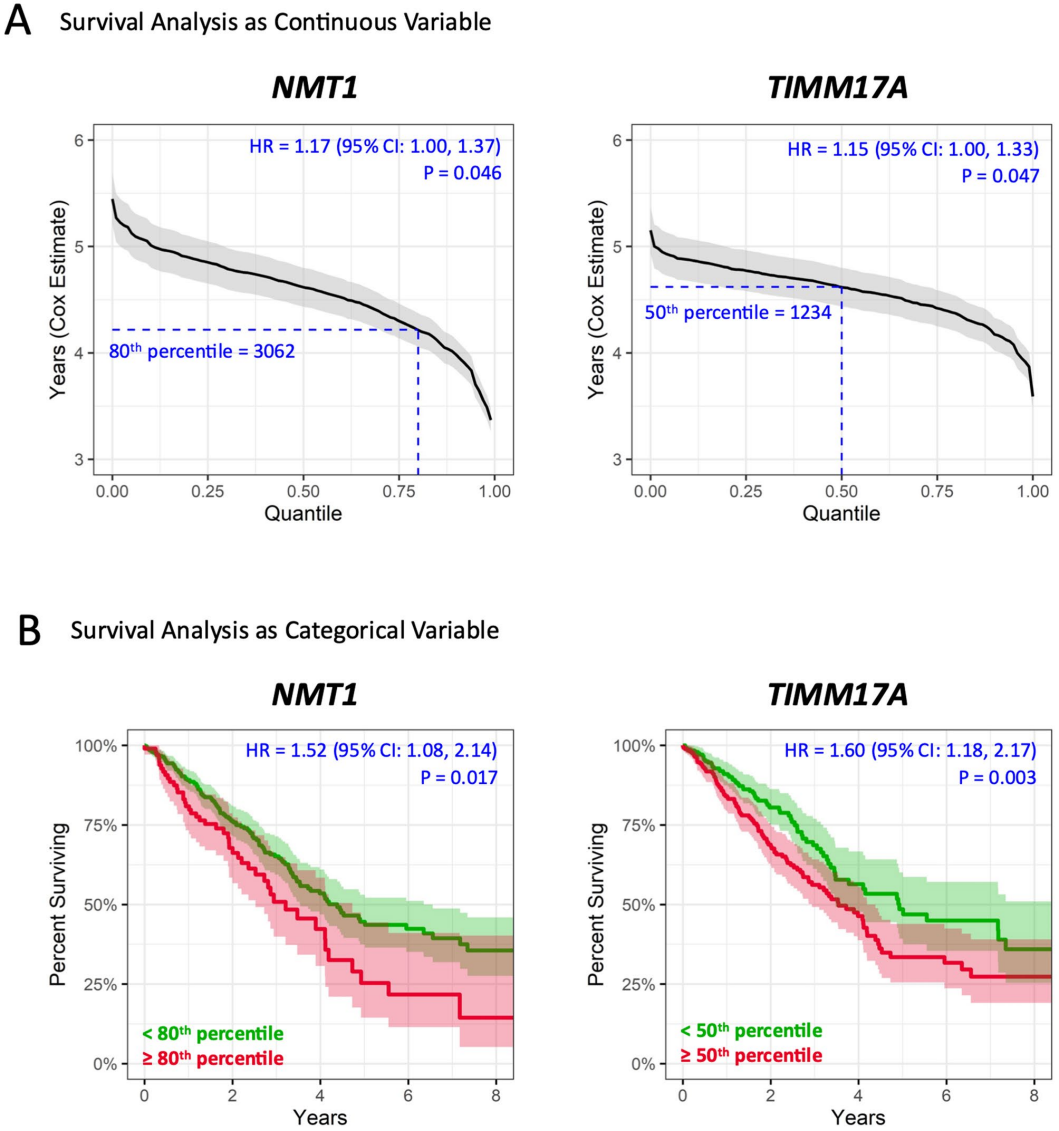
High expression of *NMT1* or *TIMM17A* was significantly associated with worse overall survival in TCGA-LUAD. TCGA-LUAD (Lung Adenocarcinoma) RNA-seq expression for *NMT1* and *TIMM17A* (normalized count) were queried from the Broad GDAC Firehose standardized pipelines. Hazard ratios (HR), their 95% confidence intervals (CI), and P-values were calculated using a cox regression model adjusted for age and gender. (**A**) Analyzed as a continuous linear variable: RMS curves (restricted mean survival) shows the cox model estimated overall survival years (y-axis) and 95% confidence band associated with nth quantile expression of reported gene (x-axis). Dashed lines report the percentile cutoffs used and the equivalent count values used to split the into dichotomous categories. (**B**) Analyzed as a dichotomous categorical variable Kaplan-Meier survival curves comparing the percent surviving (y-axis) over time (years, x-axis) between group with low expression (green) versus high expression (red). Shading in respective colors show the 95% confidence interval.

Pursuing the idea that *NMT1-TIMM17A* is a novel vulnerability axis of lung carcinomas with *STK11* and/or *KEAP1* mutations in a *KRAS* mutant background, we tested their association to OS in the TCGA-LUAD cohort using Cox-regression models. These initial models adjusted for age and gender show that the expression of *NMT1* and *TIMM17A* were each associated with reduced OS. Analyzed as a continuous variable, a one standard deviation increase in *NMT1* (log-normalized count) was associated with 17% higher mortality rate (*P* = 0.046, HR = 1.17 [95% CI 1.00, 1.37]) and *TIMM17A* was associated with 15% higher mortality rate (*P* = 0.047, HR = 1.15 [95% CI 1.00, 1.33]) (Fig. 1A); the RMS curve shows that the Cox-model estimated years of survival decreases (y-axis) as the percentile of gene expression increases (x-axis).

Next, for easier interpretability, normalized count values were grouped into categorizes of high versus low gene expression based on an optimal cutoff determined by log-rank tests evaluated at every 10 percentiles (*NMT1* cutoff: 80th percentile or 3062 count; *TIMM17A* cutoff: 50th percentile or 1234 count). Those in the group with high *NMT1* had 52% higher mortality rate compared to the low group (*P* = 0.017, HR = 1.52 [95% CI 1.08, 2.14]), and those with high *TIMM17A* had 60% higher mortality rate compared to the low group (*P* = 0.003, HR = 1.60 [95% CI 1.18, 2.17]) (Fig. 1B).

### Individual and combined effect of NMT1 and TIMM17A on OS, DSS, and DFS

Having shown that *NMT1* and *TIMM17A* were each associated with worse OS, further models tested their individual and combined association to additional clinical endpoints such as DSS and DFS. Based on the same dichotomization thresholds as for the OS models above, high *TIMM17A* was also associated with reduced DSS and DFS (*P* = 0.023 and 0.030 respectively), whereas *NMT1* showed no association to either DSS or DFS (Table 1).

**Table 1:** Association between *NMT1*, *TIMM17A*, and the combination of the two on LUAD outcome data. Hazard ratios and confidence intervals for overall survival, disease specific survival, and disease-Free survival for the various categorical splits (rows). Whereas *TIMM17A* was independently associated with overall survival, disease specific survival, and disease-Free survival, *NMT1* was only associated with overall survival. The presence of high *NMT1* together with high *TIMM17A* expression significantly had a significant association with worse OS (*P* = 0.020 as compared to *TIMM17A* alone).

	Overall survival *n* = 486, n-event 174		Disease specific survival *n* = 446, n-event = 101		Disease free survival *n* = 412, n-event = 175	
	Hazard rate (95% CI)	P value	Hazard rate (95% CI)	P value	Hazard rate (95% CI)	P value
TIMM17A						
Low	Reference	0.003	Reference	0.023	Reference	0.030
High	1.59 (1.17, 2.17)		1.60 (1.07, 2.40)		1.40 (1.03, 1.91)	
NMT1						
Low	Reference	0.017	Reference	0.316	Reference	0.583
High	1.52 ( 1.08, 2.14)		1.27 (0.79, 2.07)		1.12 (0.76, 1.64)	
Combined						
Low TIMM17A, Low NMT1	Reference	< 0.001	Reference	0.023	Reference	0.049
High TIMM17A, High NMT1	2.43 (1.52, 3.89)		2.10 (1.11, 3.99)		1.71 (1.00, 2.91)	
Combined						
Others (Low–Low; Low–High; High–Low)	Reference	0.001	Reference	0.040	Reference	0.135
High TIMM17A, High NMT1	1.99 (1.32, 2.99)		1.82 (1.03, 3.22)		1.45 (0.89, 2.35)	
P value - added NMT1 effect	0.020		0.311		0.667	
P value - NMT:TIMM17A interaction	0.868		0.429		0.671	

In combination, the categories of *NMT1* and *TIMM17A* expression had no significant interaction effect on clinical endpoints, meaning their effect on outcome is not modified by or dependent on each other (*P* = 0.87, 0.43, 0.67 for OS, DSS, and DFS respectively). Testing *NMT1* alongside *TIMM17A* in a Cox proportional hazards model showed improve fit for OS (*P* = 0.020), indicating that the two genes were independently associated with worse OS (Table 1). To explore their combination further, we tested *NMT1* effect on survival across low versus high stratification of *TIMM17A* (Supplementary Fig. S1). We found that *NMT1* was significantly associated with worse OS in the subgroup with also high *TIMM17A* (*P* = 0.016; HR = 1.71 [95% CI 1.10, 2.64]), but not in the group with low *TIMM17A* (*P* = 0.33; HR = 1.32 [95% CI 0.75, 2.33]). The effect of *NMT1* on survival was pronounced in the high expression stratum of *TIMM17A*, where it was significantly associated with worse survival. However, the effect size was not different between the two strata, thus the non-statistically significant interaction test results above. Significant association in only one of the strata could be attributed to either loss of statistical power with stratification, confounding variables within one stratum (e.g. *KRAS/KEAP1/STK11* mutations with *TIMM17A*), or possibly that *NMT1* association with worse overall survival also depends on having high *TIMM17A*. Overall, this suggests that high *NMT1* and high *TIMM17A* each contribute additively to the risk of death; *NMT1* association with worse OS is pronounced when *TIMM17A* is also elevated.

**Fig. 2:**
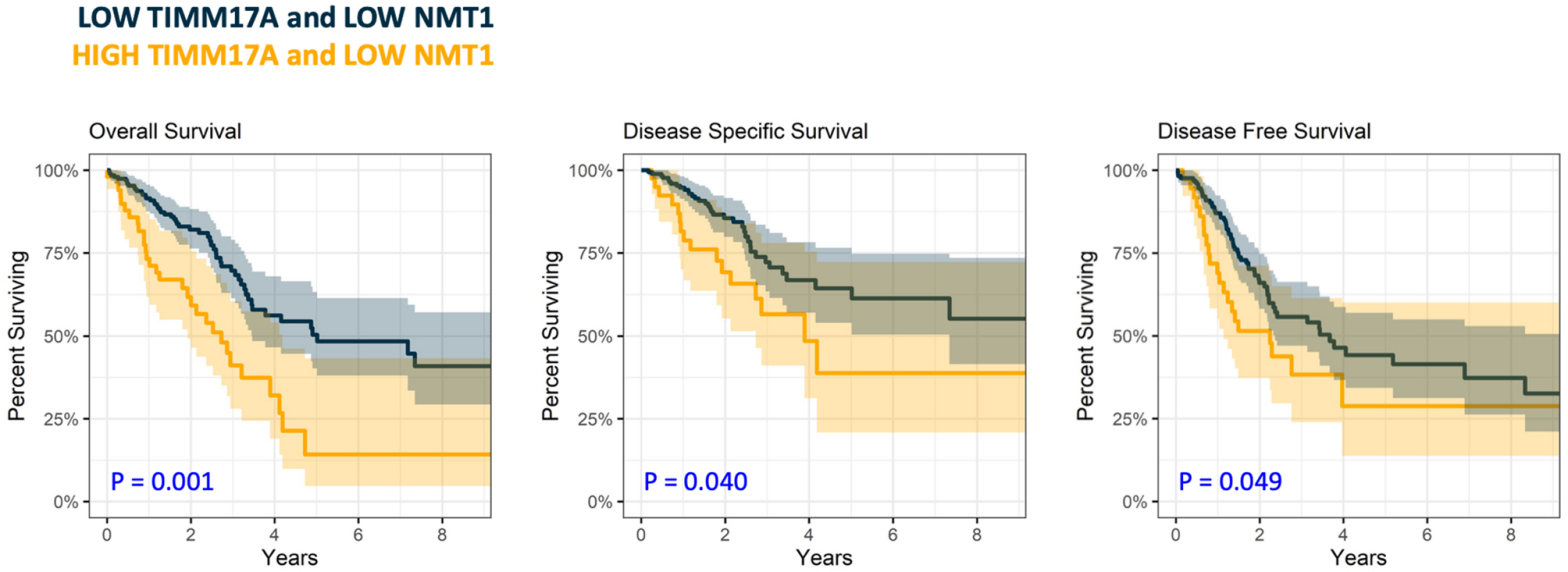
Elevated co-expression of *TIMM17A* and *NMT1* associates with worse OS, DSS and DFS. Compared to the group with both Low *TIMM17A* and Low *NMT1* (Black), those with both High *TIMM17A* and High *NMT1* (Yellow) showed worse overall survival (Left), disease-specific survival (Middle), and disease-free survival (Right) in LUAD. Shading with respective coloring displays 95% confidence interval for percent surviving (y-axis) across time (x-axis: years). P-values are calculated from initial cox regression models adjusted for age and gender with hazard rates detailed in Table 1. *NMT1* was split at the 80th percentile and *TIMM17A* was split at the 50th percentile into dichotomous categorical variables based on an optimal cutoff tested at every 10 percentiles and their assessment as continuous variables in Fig. 1.

Next, we leveraged the improved fit from combining *NMT1* and *TIMM17A*, assessing their effect together on OS, DSS, and DFS. Those with high expression of both *NMT1* and *TIMM17A* (High–High) had worse OS, DSS, and DFS compared to those with low expression of both (Low–Low) (Fig. 2) (P: <0.001, 0.023, and 0.049 respectively) (HR and 95% CI: 2.43 [1.52, 3.89], 2.10 [1.11, 3.99], and 1.71 [1.00, 2.91] respectively). Thus, patients with high co-expression of *NMT1* and *TIMM17A* had worse OS, DSS, and DFS when compared to those with low co-expression of *NMT1* and *TIMM17A*.

### Building multivariable cox regression model

**Fig. 3:**
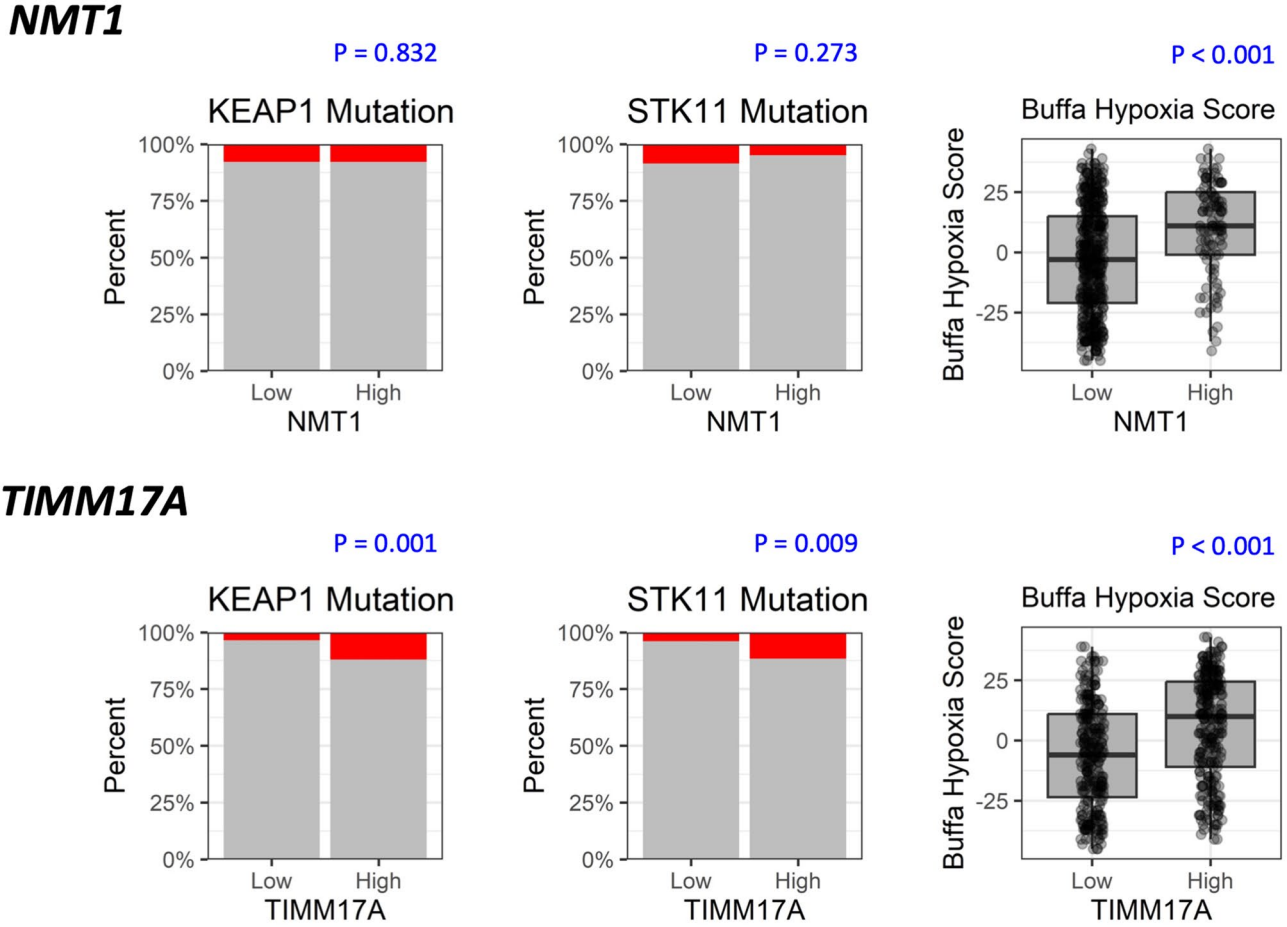
Association between different factors of interest with *TIMM17A* and *NMT1*. Potentially confounding variables would be those unevenly distributed between *TIMM17A-NMT1*, and also associated with OS. High *TIMM17A* was associated with higher incidence of *KEAP1* mutation (*P* < 0.001), *STK11* mutation (*P* = 0.002), and Buffa hypoxia score (*P* < 0.001). *NMT1* was also associated with higher hypoxia score (*P* < 0.001). Buffa hypoxia score was associated with worse OS (*P* < 0.001). *KEAP1* and *STK11* shows trend towards association to OS (*P* = 0.114 and 0.090 respectively).

We analyzed the composition of the TCGA-LUAD cohort to determine whether there were any factors confounding the *TIMM17A-NMT1* effect on outcome. We explored the distribution of demographics, mutation profiles, and clinical factors across low versus high levels of *TIMM17A* and *NMT1*, and also assessed how these variables might independently be associated with OS (Table 2). Potentially confounding variables would be those unevenly distributed between *TIMM17A-NMT1*, and also associated with OS. A higher percentage of women had low *TIMM17A* expression versus high (61.1% vs. 46.5%, *P* = 0.001), though the gender variable was not significantly associated with overall survival. Smoking shows trend towards association with *TIMM17A* (*P* = 0.093) and was associated with *NMT1* (*P* = 0.018); however, smoking was not significantly associated with overall survival. Fraction mutation altered (percentage of genome affected by copy number gains or losses) was associated with high *TIMM17A* and *NMT1* (*P* = 0.022 and 0.002 respectively); however, it was not associated with overall survival (*P* = 0.53). High *TIMM17A* was associated with higher incidence of *KEAP1* mutation (*P* = 0.001), *STK11* mutation (*P* = 0.009), and associated with having *KEAP1* and/or *STK11* along with *KRAS* mutation (*P* = 0.009). *STK11* mutation trended with poor OS (*P* = 0.090). Consistent with previous studies, *STK11* mutation in combination with *KRAS* mutation was associated with worse OS (*P* = 0.047). Hypoxia score (gene signature score calculated from transcriptomic data) may be a confounder, as it was higher with high *TIMM17A* and high *NMT1* in addition to being associated with worse overall survival (all *P* < 0.001) (Fig. 3). Interestingly, a higher Karnofsky Performance Scale at diagnosis was associated with improved survival but was unexpectedly associated with high *NMT1*.

**Table 2:** Demographic, mutation profiles, and clinical factors associated with *NMT1*, *TIMM17A*, and overall survival. Distribution of demographics, mutation profiles, and clinical factors across low versus high levels of *TIMM17A* and *NMT1*, and their association to overall survival. Comparing the distribution of continuous variables between groups (e.g. age distribution in high versus low *TIMM17A*) was performed using student t-test. Comparing the distribution of categorical variables between groups (e.g. gender distribution in high versus low *TIMM17A*) was performed using Fisher’s exact test. Highlighted in bold are *P* < 0.10 and in Italic are *P* < 0.05.

	TIMM17A Low	TIMM17A High	Pval	NMT1 Low	NMT1 High	Pval	Overall Survival	Pval
Demographics								
Age	65.0 ± 10.3	65.5 ± 9.6	0.619	65.0 ± 10.1	66.2 ± 9.5	0.278	1.09 (0.90, 1.31)	0.382
Gender								
Female	157 (61.1%)	120 (46.5%)		221 (53.6%)	56 (54.4%)		Reference	
Male	100 (38.9%)	138 (53.5%)	*0.001*	191 (46.4%)	47 (45.6%)	0.732	1.10 (0.81, 1.48)	0.543
Race								
White	190 (85.6%)	187 (87.4%)		306 (86.4%)	71 (86.6%)		Reference	
Asian	5 (2.3%)	2 (0.9%)		5 (1.4%)	2 (2.4%)		0.72 (0.43, 1.19)	0.473
Black	27 (12.2%)	25 (11.7%)	0.636	43 (12.1%)	9 (11%)	0.791	0.49 (0.07, 3.48)	0.203
Smoking category								
Non-Smoker	36 (15.3%)	30 (13%)		47 (12.6%)	19 (20%)		Reference	
Reform ≤ 15	69 (29.2%)	91 (39.4%)		126 (33.9%)	34 (35.8%)		0.83 (0.50, 1.38)	0.471
Reform > 15	65 (27.5%)	62 (26.8%)		112 (30.1%)	15 (15.8%)		0.90 (0.54, 1.50)	0.697
Current	66 (28%)	48 (20.8%)	**0.093**	87 (23.4%)	27 (28.4%)	*0.018*	0.96 (0.60, 1.54)	0.863
Year of smoking onset (smokers)	43.2 ± 12.0	44.7 ± 11.8	0.288	43.8 ± 12.0	44.3 ± 11.5	0.986	0.99 (0.80, 1.23)	0.927
Pack years (smokers)	41.1 ± 29.4	43.2 ± 25.3	0.470	41.2 ± 28.0	46.2 ± 24.6	0.154	1.09 (0.90, 1.31)	0.382
ECOG performance status								
0-Fully Active	50 (47.2%)	38 (37.3%)		68 (40.2%)	20 (51.3%)		Reference	
1-Restricted Strenuous Activity	45 (42.5%)	51 (50%)		82 (48.5%)	14 (35.9%)		1.16 (0.73, 1.85)	0.539
2-Ambulatory and Full Selfcare	9 (8.5%)	12 (11.8%)		17 (10.1%)	4 (10.3%)		1.64 (0.71, 3.78)	0.243
3-Limited Selfcare	2 (1.9%)	1 (1%)	0.465	2 (1.2%)	1 (2.6%)	0.366	2.32 (0.55, 9.76)	0.250
Karnofsky performance score	81.9 ± 25.3	75.4 ± 30.6	0.189	76.2 ± 29.7	90.4 ± 14.0	*< 0.001*	0.68 (0.55, 0.83)	*< 0.001*
FEV1% pre-broncholiator	81.7 ± 24.7	79.5 ± 22.5	0.481	81.0 ± 22.6	80.0 ± 28.5	0.833	0.80 (0.62, 1.03)	**0.082**
FEV1% post-broncholiator	80.3 ± 26.9	80.0 ± 21.3	0.940	81.3 ± 23.5	73.8 ± 29.1	0.290	0.77 (0.55, 1.07)	0.119
FEV1/FVC pre-broncholiator	82.1 ± 18.9	75.9 ± 17.2	*0.020*	80.5 ± 18.4	76.1 ± 18.4	0.218	0.98 (0.73, 1.32)	0.885
FEV1/FVC post-broncholiator	80.7 ± 21.5	74.9 ± 20.1	0.143	79.3 ± 19.9	70.2 ± 27.7	0.272	0.69 (0.48, 1.00)	**0.052**
Carbon monoxide diffusion DLCO	75.4 ± 23.0	70.4 ± 22.5	0.126	3.7 ± 23.1	71.8 ± 21.9	0.641	0.80 (0.59, 1.08)	0.147
Mutations								
KRAS								
No	210 (86.8%)	204 (84.3%)		328 (84.3%)	86 (90.5%)		Reference	
Yes	32 (13.2%)	38 (15.7%)	0.518	61 (15.7%)	9 (9.5%)	0.144	0.99 (0.65, 1.51)	0.962
KEAP1								
No	233 (96.3%)	213 (88%)		359 (92.3%)	87 (91.6%)		Reference	
Yes	9 (3.7%)	29 (12%)	*0.001*	30 (7.7%)	8 (8.4%)	0.832	1.44 (0.92, 2.25)	0.114
STK11								
No	232 (95.9%)	216 (89.3%)		357 (91.8%)	91 (95.8%)		Reference	
Yes	10 (4.1%)	26 (10.7%)	*0.009*	32 (8.2%)	4 (4.2%)	0.273	1.49 (0.94, 2.35)	**0.090**
KRAS and KEAP1/STK11								
No	237 (97.9%)	224 (92.6%)		369 (94.9%)	92 (96.8%)		Reference	
Yes	5 (2.1%)	18 (7.4%)	*0.009*	20 (5.1%)	3 (3.2%)	0.592	1.78 (1.01, 3.14)	*0.047*
EGFR								
No	238 (92.6%)	242 (93.8%)		386 (93.7%)	94 (91.3%)		Reference	
Yes	19 (7.4%)	16 (6.2%)	0.592	26 (6.3%)	9 (8.7%)	0.426	1.20 (0.71, 2.04)	0.492
ALK translocation status								
No	91 (84.3%)	108 (86.4%)		161 (85.2%)	38 (86.4%)		Reference	
Yes	17 (15.7%)	17 (13.6%)	0.711	28 (14.8%)	6 (13.6%)	1.000	1.84 (1.02, 3.31)	*0.042*
Aneuploidy score	14.7 ± 8.3	15.4 ± 7.4	0.372	14.3 ± 7.9	18.1 ± 7.0	*< 0.001*	1.06 (0.91, 1.23)	0.454
Buffa hypoxia score	-7.3 ± 21.3	5.6 ± 22.0	*< 0.001*	-3.3 ± 22.7	9.2 ± 19.5	*< 0.001*	1.42 (1.21, 1.67)	*< 0.001*
Ragnum hypoxia score	-1.2 ± 13.5	5.5 ± 14.2	*< 0.001*	0.4 ± 14.1	9.6 ± 12.3	*< 0.001*	1.34 (1.15, 1.57)	*< 0.001*
Winter hypoxia score	-3.7 ± 25.4	9.8 ± 24.2	*< 0.001*	0.1 ± 25.7	15.1 ± 22.0	*< 0.001*	1.41 (1.21, 1.66)	*< 0.001*
Fraction genome altered	0.24 ± 0.20	0.28 ± 0.18	*0.022*	0.25 ± 0.19	0.32 ± 0.18	*0.002*	1.05 (0.91, 1.21)	0.525
Total mutation	283.0 ± 326.7	294.9 ± 246.2	0.655	274.1 ± 266.4	349.9 ± 363.4	**0.061**	0.97 (0.83, 1.14)	0.729
Total mutation burden (nonsynonymous) per megabase	10.3 ± 12.1	10.5 ± 9.4	0.804	9.9 ± 10.1	12.7 ± 13.4	**0.063**	0.94 (0.80, 1.11)	0.463
MSI MANTIS score	0.318 ± 0.025	0.314 ± 0.025	*0.048*	0.316 ± 0.026	0.316 ± 0.022	0.981	1.05 (0.89, 1.24)	0.572
MSI sensor score	0.149 ± 0.559	0.144 ± 0.419	0.918	0.149 ± 0.537	0.139 ± 0.256	0.818	1.15 (1.00, 1.31)	*0.044*
Clinical factors								
Tissue weight	190.2 ± 129.5	196.2 ± 176.5	0.833	201.5 ± 157.1	130.7 ± 111.7	*0.047*	1.32 (1.02, 1.70)	*0.032*
Prior cancer diagnosis occurrence								
No	200 (82.6%)	198 (81.8%)		322 (82.8%)	76 (80%)		Reference	
Yes	42 (17.4%)	44 (18.2%)	0.905	67 (17.2%)	19 (20%)	0.550	1.45 (0.98, 2.16)	**0.063**
Primary tumor location								
L-Lower	37 (15.9%)	33 (13.9%)		55 (14.7%)	15 (15.6%)		Reference	
L-Upper	60 (25.8%)	56 (23.6%)		87 (23.3%)	29 (30.2%)		0.89 (0.54, 1.46)	0.643
R-Lower	45 (19.3%)	47 (19.8%)		70 (18.7%)	22 (22.9%)		1.17 (0.71, 1.92)	0.539
R-Middle	10 (4.3%)	10 (4.2%)		17 (4.5%)	3 (3.1%)		0.75 (0.26, 2.16)	0.593
R-Upper	81 (34.8%)	91 (38.4%)	0.918	145 (38.8%)	27 (28.1%)	0.286	0.91 (0.58, 1.45)	0.706
Location lung perenchyma								
Central	26 (34.7%)	37 (38.1%)		54 (39.7%)	9 (25%)		Reference	
Peripheral	49 (65.3%)	60 (61.9%)	0.750	82 (60.3%)	27 (75%)	0.122	1.10 (0.68, 1.77)	0.693
Pathological stage								
I	135 (56.5%)	119 (50%)		212 (55.5%)	42 (44.2%)		Reference	
II	58 (24.3%)	58 (24.4%)		89 (23.3%)	27 (28.4%)		1.99 (1.36, 2.90)	*< 0.001*
III	36 (15.1%)	45 (18.9%)		62 (16.2%)	19 (20%)		3.25 (2.22, 4.76)	*< 0.001*
IV	10 (4.2%)	16 (6.7%)	**0.069**	19 (5%)	7 (7.4%)	**0.073**	3.43 (1.98, 5.96)	*< 0.001*
T Stage								
T1	94 (39.2%)	70 (29%)		138 (35.7%)	26 (27.7%)		Reference	
T2	118 (49.2%)	135 (56%)		206 (53.2%)	47 (50%)		1.40 (0.98, 2.01)	**0.067**
T3	23 (9.6%)	22 (9.1%)		28 (7.2%)	17 (18.1%)		2.83 (1.68, 4.76)	*< 0.001*
T4	5 (2.1%)	14 (5.8%)	*0.012*	15 (3.9%)	4 (4.3%)	*0.032*	2.96 (1.56, 5.62)	*0.001*
N Stage								
n0	167 (71.4%)	146 (61.3%)		257 (67.8%)	56 (60.2%)		Reference	
n1	34 (14.5%)	53 (22.3%)		67 (17.7%)	20 (21.5%)		2.19 (1.53, 3.13)	*< 0.001*
n2	31 (13.2%)	39 (16.4%)		53 (14%)	17 (18.3%)		2.91 (1.99, 4.27)	*< 0.001*
n3	2 (0.9%)	0 (0%)	*0.045*	2 (0.5%)	0 (0%)	0.177	NA	
M Stage								
m0	147 (94.2%)	171 (91.4%)		251 (93.3%)	67 (90.5%)		Reference	
m1	9 (5.8%)	16 (8.6%)	0.406	18 (6.7%)	7 (9.5%)	0.449	1.98 (1.16, 3.40)	*0.013*
Radiation therapy								
No	196 (87.5%)	181 (84.6%)		311 (87.4%)	66 (80.5%)		Reference	
Yes	28 (12.5%)	33 (15.4%)	0.409	45 (12.6%)	16 (19.5%)	0.113	1.93 (1.32, 2.81)	*0.001*
Postoperative adjuvant therapy								
No	68 (79.1%)	49 (72.1%)		103 (77.4%)	14 (66.7%)		Reference	
Yes	18 (20.9%)	19 (27.9%)	0.346	30 (22.6%)	7 (33.3%)	0.283	1.67 (0.88, 3.15)	0.116

To investigate the effect of these various factors, we iteratively added and removed terms from our regression model to assess how each may relate to OS (Table 3). Our initial model included both gender and age based on common practice, though neither significantly contributed to a better model fit (*P* = 0.78 and 0.45 respectively). The next set of models included tumor pathological staging (known confounder) to evaluate *NMT1* and *TIMM17A* as independent factors to predict survival. We found that whether stage was added as an ordinal variable or a categorical variable (I/II vs. III/IV), high expression of both *TIMM17A* and *NMT1* remained associated with reduced OS. These findings suggest that the prognostic effects of *TIMM17A *and *NMT1 *on survival are not explained by tumor stage and may serve as independent markers for poor prognosis. The fourth model with hypoxia score confounded with *TIMM17A* and *NMT1* with some multicollinearity, and the two genes of interest were no longer statistically significant. Adding hypoxia score resulted in redundancy in the information each variable provides to the model, suggesting that *TIMM17A* and *NMT1* are either related to or influenced by hypoxia score. Much of the prognostic effect of *TIMM17A* and *NMT1* is explained by the hypoxic status of the tumor. Finally, the complete model included *KRAS/STK11/KEAP1* mutation to isolate the effect of *TIMM17A* and *NMT1* on survival. From Table 2, the prevalence of *KRAS* co-mutation with *KEAP1*/*STK11* were associated with high *TIMM17A* (*P* = 0.009) as well as worse OS (*P* = 0.047), and therefore is a potential confounder. The complete model showed that both high *TIMM17A* and high *NMT1* were still associated with worse OS (*TIMM17A*: *P* = 0.005, HR = 1.56 [95% CI 1.14, 2.14] and *NMT1*: *P* = 0.023, HR = 1.51 [95% CI 1.06, 1.14]). *KRAS*,* STK11*,* and KEAP1* mutations, on the other hand, were not associated with survival. While *STK11* mutation did not reach statistical significance in the complete model (*P* = 0.069), it showed a trend towards worse OS, indicating that STK11 mutation may still have a potential role in influencing survival outcomes. Due to low frequency of these mutations (e.g. *n* = 36 for *STK11*), the analysis lacks statistical power to determine whether these mutations or the combination of them mediates the association between *TIMM17A*, *NMT1*, and survival. Ultimately, the full model adjusting for potential confounders showed that high expression of *TIMM17A* and of *NMT1* were independently linked to worse OS. The combination of high-high expression of *TIMM17A* and *NMT1* had 240% higher rate of death compared to those with low-low expression (*P* < 0.001, HR = 2.40 [95% CI 1.51, 3.85], not shown).

**Table 3:** Building multivariable Cox regression with *TIMM17A *and *NMT1* on overall survival. The complete model adjusted for gender, age, tumoral stage, and presence of *KRAS*,* STK11*,* KEAP1* mutations showed that both high *TIMM17A* and high *NMT1* were associated with worse OS (*TIMM17A*: *P* = 0.005, HR = 1.56 [95% CI: 1.14, 2.14] and *NMT1*: *P* = 0.023, HR = 1.51 [95% CI: 1.06, 1.14]).

	Overall survival HR (95% CI)	*P* value
Model 1: Initial model		
TIMM17A: Low vs. high	1.60 (1.17, 2.18)	0.003**
NMT1: Low vs. high	1.53 (1.08, 2.15)	0.016*
Gender: Female vs. male	1.01 (0.74, 1.37)	0.942
Age	1.01 (0.99, 1.02)	0.540
*n* = 484, n-events = 174		

Adding stage	Overall survival HR (95% CI)	*P* value
Model 2: Ordinal stage		
TIMM17A: Low vs. high	1.48 (1.10, 1.99)	0.010**
NMT1: Low vs. high	1.48 (1.07, 2.07)	0.019*
Stage: Ordinal	1.67 (1.45, 1.92)	< 0.001***
Model 3: Stage I/II vs. III/IV		
TIMM17A: Low vs. high	1.48 (1.10, 1.99)	0.010*
NMT1: Low vs. high	1.57 (1.12, 2.18)	0.008**
Stage: I/II vs. III/IV	2.68 (1.96, 3.64)	< 0.001***
*n* = 498, n-events = 181		

Adding hypoxia score	Overall Survival HR (95% CI)	*P* value
Model 4: Hypoxia score		
TIMM17A: Low vs. high	1.30 (0.96, 1.77)	**0.092**
NMT1: Low vs. high	1.35 (0.96, 1.91)	**0.084**
Stage: I/II vs. III/IV	2.56 (1.87, 3.49)	< 0.001***
Buffa hypoxia score (scaled)	1.29 (1.10, 1.52)	0.002**
*n* = 493, n-events = 179		

	Overall survival HR (95% CI)	*P* value
Complete model		
TIMM17A: Low vs. high	1.56 (1.14, 2.14)	0.005**
NMT1: Low vs. high	1.51 (1.06, 2.14)	0.023*
Gender: Female vs. male	0.92 (0.68, 1.26)	0.608
Age	1.06 (0.90, 1.24)	0.495
Stage: I/II vs. III/IV	2.65 (1.92, 3.65)	< 0.001***
KRAS	0.86 (0.54, 1.37)	0.533
KEAP1	0.96 (0.59, 1.57)	0.866
STK11	1.62 (0.96, 2.72)	0.069
*n* = 477, n-events = 172		

### Validating results in OncoSG ever-smokers

**Fig. 4:**
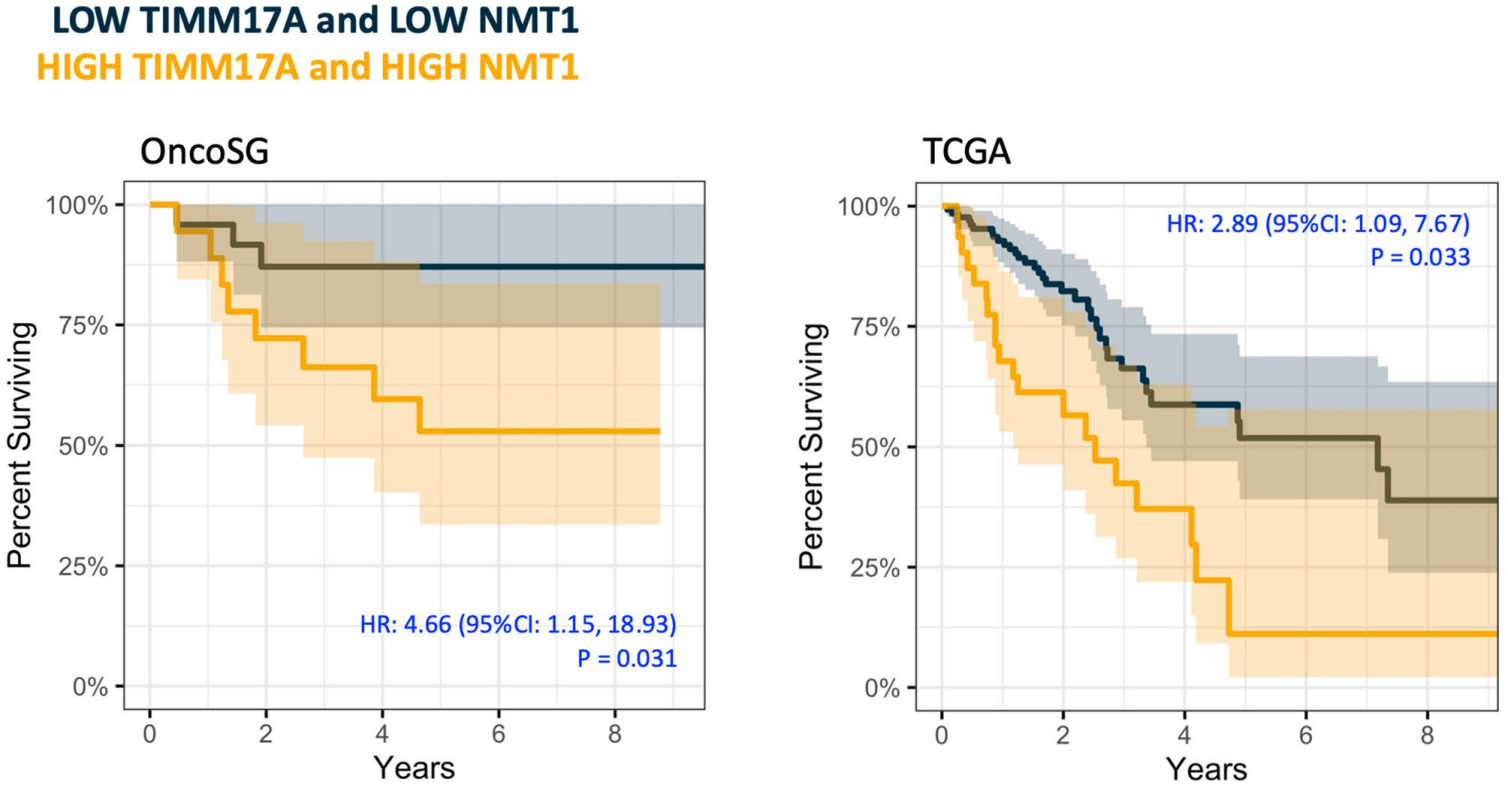
Elevated co-expression of *TIMM17A* and *NMT1* associates with worse OS in the smoking subset of the OncoSG and TCGA dataset.

Since the TCGA cohort comprise of 85% ever-smokers, results were validated using the ever-smokers of the OncoSG database. Similar to the findings in the TCGA, the OncoSG dataset also showed that higher *NMT1* and higher *TIMM17A* were each associated with worse overall survival after adjusting for age, gender, and tumor stage (Supplementary Fig. S2; *NMT1*: *P* = 0.033, HR = 2.89 [95% CI 1.09, 7.67] and *TIMM17A*: *P* = 0.023, HR = 3.67 [95% CI 1.20, 11.20]). Likewise, when we leveraged the improved fit from combining *NMT1* and *TIMM17A*, high-high compared to low-low expression of *NMT1* and *TIMM17A* together was associated with worse OS for ever-smokers in both the OncoSG and TCGA datasets, even after adjusting for age, gender, and tumor stage (Fig. 4; OncoSG: *P* = 0.031, HR = 4.66 [95% CI 1.15, 18.93] and TCGA: *P* = 0.033, HR = 2.89 [1.09, 7.67]).

## Discussion

Here, we report that the combination of both high *NMT1* and high *TIMM17A* was associated with poor prognosis (worse OS, DSS, and DFS) in lung adenocarcinoma. We validate previous reports that *NMT1*^11,13^ and *TIMM17A*^13^ are each independently associated with reduced OS. We additionally found that *TIMM17A* expression is associated with reduced DSS and DFS. Interestingly, *NMT1* was associated with reduced OS, but only in the subset with also high *TIMM17A* (which is also the subset associated with higher prevalence of *STK11* and *KEAP1* mutations). This is consistent with our previous observations that *NMT1* inhibition is particularly effective against lung carcinomas with *STK11* and/or *KEAP1* mutations in a *KRAS* mutant background^12^, and that the sensitivity of lung carcinoma cells to NMT inhibition correlates with TIM17A dependency^12^. We found that assessing both *NMT1* and *TIMM17A* together revealed a pronounced reduction in OS, DSS, and DFS. Co-overexpression of *NMT1* and *TIMM17A* is a predictor of poor survival and a potentially targetable pathway to treat *KRAS STK11* and *KEAP1*-mutant NSCLC.

Given the TCGA-LUAD cohort predates and is not confounded by the incorporation of ICI in treatment of NSCLC, our survival analysis could not investigate how immunotherapy might alter the association between *NMT1* and *TIMM17A* and survival. Further limitation is that the cohort mostly consists of Caucasian from United States, who are more likely to have a history of current or former tobacco use (representing 85% of the total cohort). As such, the TCGA cohort’s tumor samples are expected to have higher tumor mutational burden and the total number of mutations in the tumor are strongly associated with smoking. This and other population differences may contribute to differences at the mRNA expression levels, which are relative to each study. The results were validated with the smoking subset of the OncoSG dataset, which comprised of individuals from East Asia; however, studying a larger non-smoking Caucasian cohort as well as studying other ethnic groups will help widen the scope and generalizability of these results. Lastly, the number of mutations of *KRAS*, *STK11*, and *KEAP1* in lung cancer were few, even in the TCGA cohort, which hinders the ability to detect the effect of these mutations on survival. An even larger sample size would be necessary to robustly assess how these mutations may mediate *TIMM17A*, *NMT1*, and outcome.

A strength of this study was in using an updated dataset as opposed to a provisional version^13^, thus having a larger sample size (*n* = 486 versus 258) with longer follow-up periods. Additionally, we show that *NMT1* was associated to overall survival not only as a categorical variable, but also as a continuous variable; splitting gene expression into dichotomous high versus low simplifies the relationship, but analysis as a continuous variable preserves the data and reveals a linear rise in risk with rise in *NMT1* expression level. Furthermore, we illustrate how assessing both *NMT1* and *TIMM17A* together shows a pronounced difference in not only OS, but also DSS and DFS. This is surprising given the fact that TIM17A is not a myristoylation target of NMT1. Lastly, our statistical model highlights the value of considering both *NMT1* and *TIMM17A* expression as independent predictors of survival that are beyond what could be modeled using pathological tumor staging. Our findings warrant further studies to understand the relevance of the novel *NMT1*-*TIMM17A* axis in the progression and treatment of *KRAS/STK11/KEAP1* NSCLC.

## Electronic supplementary material

Below is the link to the electronic supplementary material.

Supplementary Material 1

## Data Availability

The processed sequencing data and associated de-identified patient clinical data analyzed in this study are publicly available in cBioPortal for the TCGA–LUAD (PanCancer Atlas) and in the OncoSG portal. https://www.cbioportal.org/study/summary?id=luad_tcga_pan_can_atlas_2018. https://bioinfo.asia/oncosg_public/dwls/GIS031_GSK.zip.
