# Supplementary material for: Elevated co-expression of TIMM17A and NMT1 is associated with poor survival in non-small cell lung cancer
Source: Sci Rep. 2025 Oct 13;15:35597. doi: 10.1038/s41598-025-11897-9 (PMC12518850; doi:10.1038/s41598-025-11897-9)

# Elevated co-expression of TIMM17A and NMT1 is associated with poor survival in non-small cell lung cancer

Alfred A. Chan ^1^, Kamya Sankar ^2^, Karen L. Reckamp ^2^, Begoña Díaz ^*1,3,4^, Delphine J. Lee ^*1,4,5^

1. The Lundquist Institute for Biomedical Innovation at Harbor-UCLA Medical Center, Torrance, California 90502, USA

2. Department of Medicine, Cedars-Sinai Medical Center, Los Angeles, CA, USA.

3. Division of Hematology and Oncology at Harbor-UCLA Medical Center, David Geffen School of Medicine at UCLA, Los Angeles, California, USA

4. Jonsson Comprehensive Cancer Center, UCLA, Los Angeles, California, USA

5. Division of Dermatology at Harbor-UCLA Medical Center, David Geffen School of Medicine at UCLA, Los Angeles, California.

*co-senior

**Corresponding Author**

Delphine J. Lee

Chief and Program Director

Medicine/Dermatology

[delphine.lee@lundquist.org](mailto:delphine.lee@lundquist.org)

p: (424) 571-7763

THE LUNDQUIST INSTITUTE

1124 West Carson Street,

Torrance, CA 90502

[https://lundquist.org](https://urldefense.com/v3/__https:/lundquist.org__;!!KOmnBZxC8_2BBQ!wE9R1g9bjHAbqdPPJjAsHSzzzzvQfJwyjuMZhpb2VMFFeV-Al7Uz1KvyoF3slKhwnH5992M9Yz44XQI4tpx-s08nO4c$)

**Key Words:** The Cancer Genome Atlas, Lung Adenocarcinoma, NMT1, TIMM17A, Survival

# Supplemental Figure S1 – Association between high NMT1 and poor survival was dependent on also having high expression of TIMM17A.

Among patients with high *TIMM17A* (Right), those with high *NMT1* (Red) had significantly lower overall survival compared to those with low *NMT1* (Green). Among patients with Low *TIMM17A* (Left), there were no such differences in overall survival by *NMT1* expression, with high overlaps in their confidence intervals.


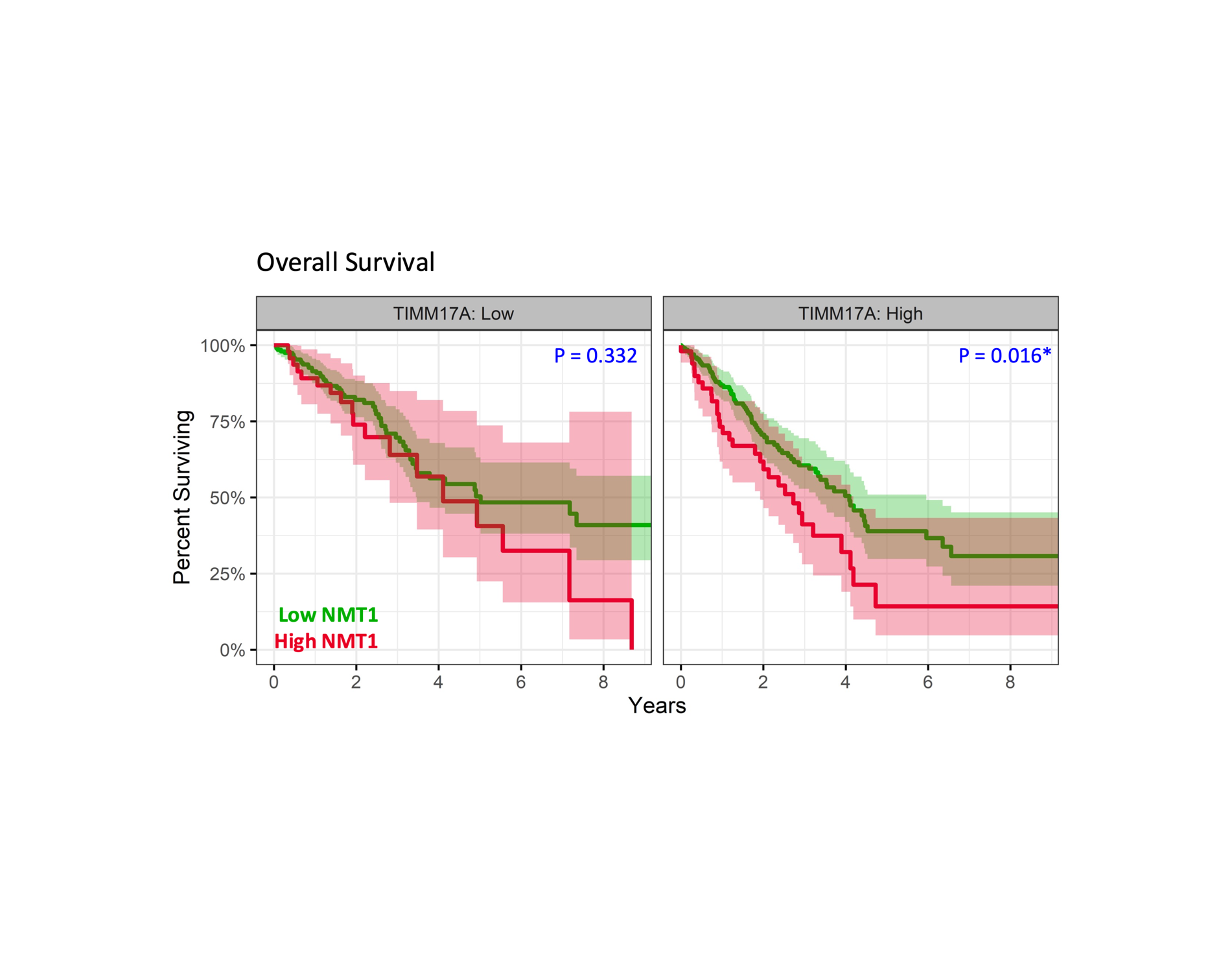


# Supplemental Figure S2 – *TIMM17A* and *NMT1* were each associated with worse overall survival in OncoSG ever-smokers

Kaplan-Meier plot comparing high (red) versus low (green) expression of *NMT1* (left) and *TIMM17A* (right) by RNAseq in the OncoSG validation dataset of ever-smokers (comparable to TCGA, which has 85% ever-smokers). Shading in their respective colors represent 95% confidence interval. Hazard Ratios (HR), their 95% confidence intervals (CI), and P-values were calculated using multivariable cox-regression models adjusted for age (mean±SD: 64.1±10.0), gender (n: Male=49, Female=13), and tumor stage (n: I=35, II=11, III=13, IV=1). High TIMM17A and high NMT1 were each associated with worse overall in the ever-smoker subset of OncoSG. *NMT1* was split at the 60^th^ percentile and *TIMM17A* was split at the 50^th^ percentile based on an optimal cutoff tested at every 10 percentiles amongst ever-smokers


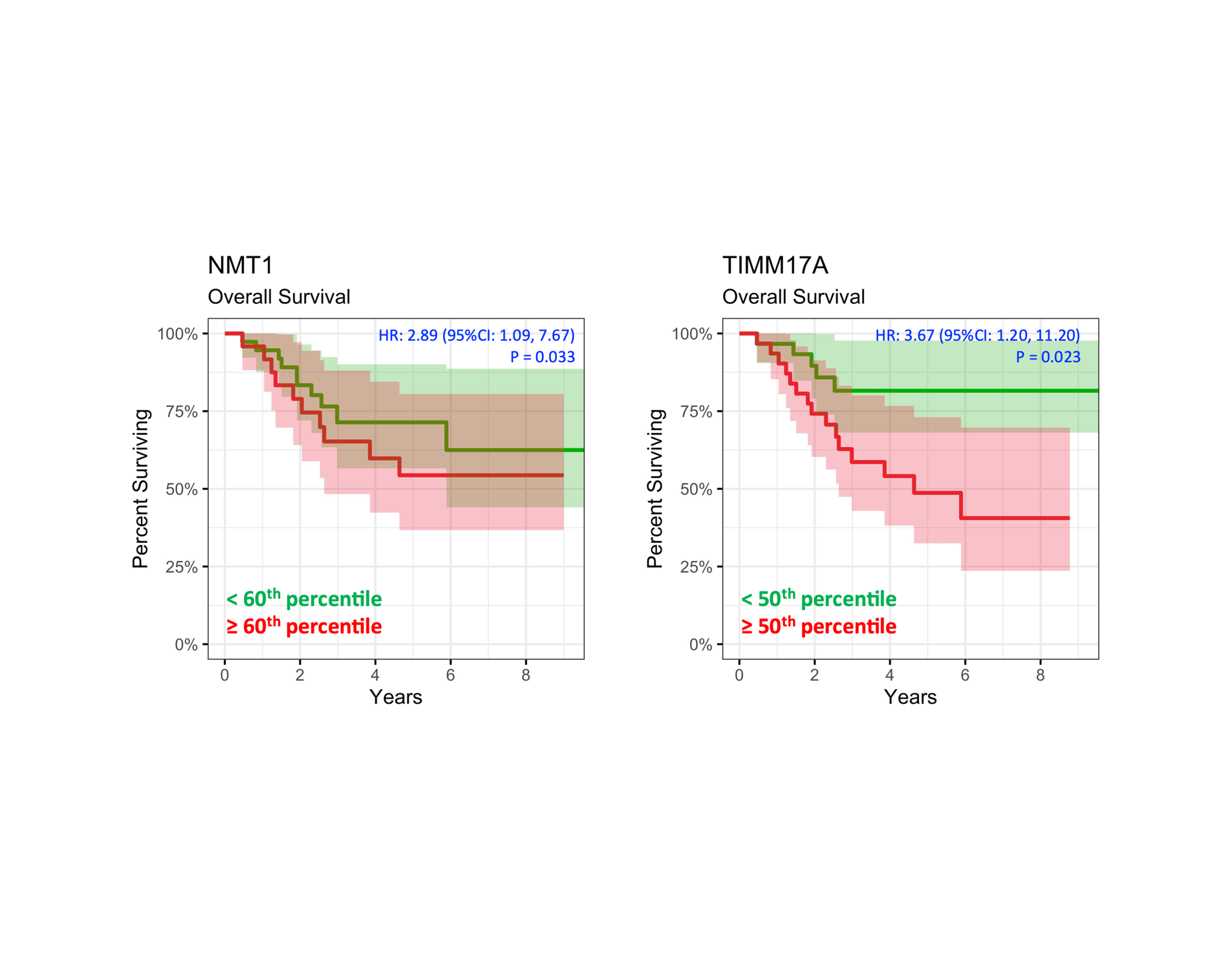

Supplement: Supplementary file 1 — Supplementary Material 1 [file 41598_2025_11897_MOESM1_ESM.docx]
